# Baicalin and baicalein from *Scutellaria baicalensis* Georgi alleviate aberrant neuronal suppression mediated by GABA from reactive astrocytes

**DOI:** 10.1111/cns.14740

**Published:** 2024-05-07

**Authors:** Juyeong Cho, Eun‐Bin Hong, Young‐Sik Kim, Jungbin Song, Yeon Ha Ju, Hyunjin Kim, Hyowon Lee, Hocheol Kim, Min‐Ho Nam

**Affiliations:** ^1^ Center for Brain Function, Brain Science Institute Korea Institute of Science and Technology (KIST) Seoul Republic of Korea; ^2^ Department of Herbology, College of Korean Medicine Woosuk University Jeonju‐si Republic of Korea; ^3^ Department of Herbal Pharmacology, College of Korean Medicine Kyung Hee University Seoul Republic of Korea; ^4^ Department of KHU‐KIST Convergence Science and Technology Kyung Hee University Seoul Republic of Korea; ^5^ Division of Bio‐Medical Science & Technology, KIST School University of Science and Technology Seoul Republic of Korea

**Keywords:** GABA, MAO‐B, neuroinflammation, reactive astrogliosis, *Scutellaria baicalensis* Georgi, tonic inhibition

## Abstract

**Aims:**

γ‐aminobutyric acid (GABA) from reactive astrocytes is critical for the dysregulation of neuronal activity in various neuroinflammatory conditions. While *Scutellaria baicalensis* Georgi (*S. baicalensis*) is known for its efficacy in addressing neurological symptoms, its potential to reduce GABA synthesis in reactive astrocytes and the associated neuronal suppression remains unclear. This study focuses on the inhibitory action of monoamine oxidase B (MAO‐B), the key enzyme for astrocytic GABA synthesis.

**Methods:**

Using a lipopolysaccharide (LPS)‐induced neuroinflammation mouse model, we conducted immunohistochemistry to assess the effect of *S. baicalensis* on astrocyte reactivity and its GABA synthesis. High‐performance liquid chromatography was performed to reveal the major compounds of *S. baicalensis*, the effects of which on MAO‐B inhibition, astrocyte reactivity, and tonic inhibition in hippocampal neurons were validated by MAO‐B activity assay, qRT‐PCR, and whole‐cell patch‐clamp.

**Results:**

The ethanolic extract of *S. baicalensis* ameliorated astrocyte reactivity and reduced excessive astrocytic GABA content in the CA1 hippocampus. Baicalin and baicalein exhibited significant MAO‐B inhibition potential. These two compounds downregulate the mRNA levels of genes associated with reactive astrogliosis or astrocytic GABA synthesis. Additionally, LPS‐induced aberrant tonic inhibition was reversed by both *S. baicalensis* extract and its key compounds.

**Conclusions:**

In summary, baicalin and baicalein isolated from *S. baicalensis* reduce astrocyte reactivity and alleviate aberrant tonic inhibition of hippocampal neurons during neuroinflammation.

## Graphical abstract

Baicalin and baicalein, the major components of *Scutellaria baicalensis*, inhibit the enzymatic activity of monoamine oxidase B (MAO‐B), thereby diminishing GABA synthesis in reactive astrocytes. This reduction in astrocytic GABA production leads to the alleviation of GABA_A_ receptor‐mediated tonic inhibition in hippocampal neurons.

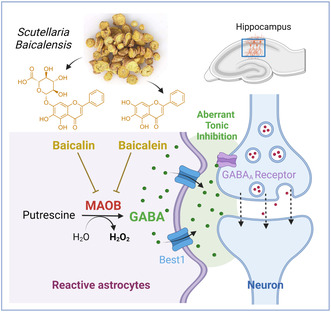

## 1 INTRODUCTION

The maintenance of homeostatic equilibrium in neuronal activity is imperative for the regulation of cerebral physiological functions. Dysregulation of neuronal activity, whether it is heightened or diminished, leads to functional problems. A growing body of evidence underscores the pivotal role of astrocytes in mediating neuronal activity. Specifically, γ‐aminobutyric acid (GABA), a widely acknowledged inhibitory neurotransmitter, can be synthesized and released tonically by astrocytes. The astrocytic GABA is particularly significant in the tonic regulation of neuronal activity. Notably, there is an excessive upregulation of astrocytic GABA synthesis during the pathological transformation of astrocytes known as reactive astrogliosis.

Reactive astrogliosis is a pathological phenomenon involving morphological and transcriptional changes in astrocytes during neuroinflammation.^1^ Among the various genes involved in this phenomenon, the monoamine oxidase B (MAO‐B) enzyme, located in the outer mitochondrial membrane of astrocytes, has emerged as a key factor for mediating the entire cascade of reactive astrogliosis.^2^ Specifically, MAO‐B mediates the excessive synthesis of γ‐aminobutyric acid (GABA) and hydrogen peroxide (H_2_O_2_) in reactive astrocytes across various brain regions.^3^, ^4^, ^5^, ^6^, ^7^ The astrocytic GABA, synthesized in an MAO‐B‐dependent manner, is tonically released and can aberrantly suppress neighboring neuronal activity, leading to tonic inhibition. Excessive tonic inhibition mediated by MAO‐B has been causally linked to memory deficits in Alzheimer's disease (AD),^4^, ^5^ motor dysfunctions in Parkinson's disease (PD),^8^ and the limited effect of rehabilitation therapy in stroke.^7^ Moreover, pharmacological blockade of MAO‐B has been reported to significantly reverse reactive astrogliosis‐mediated pathology and symptoms in rodent models of these disorders.^6^, ^7^, ^9^ Therefore, MAO‐B‐mediated neuronal suppression represents a promising target for the development of novel drugs that can treat brain disorders accompanied by neuroinflammation.

In recent years, traditional East Asian medicines, which include medicinal herbs and their compounds, have gained popularity in the clinical treatment of brain disorders due to their safety, effectiveness, and affordability. The root of *Scutellaria baicalensis* Georgi is frequently used in traditional East Asian medicine and is known for its multiple pharmacological effects, such as anti‐inflammation, antitumor, and antioxidant properties.^10^ *S. baicalensis*, commonly known as Baikal skullcap or Chinese skullcap, belongs to the flowering plant family Lamiaceae. It is indigenous to China, Korea, Mongolia, as well as the Russian Far East and Siberia regions of Russia. Previous studies have shown that the bioactive compounds of *S. baicalensis*, including baicalin, baicalein, and wogonin, exert beneficial effects in PD,^11^ attention‐deficit/hyperactive disorder (ADHD),^12^ brain injury,^13^ depression,^14^ and cognitive impairment.^15^ Although many natural compounds have limited use as neuroprotective agents due to their poor blood‐brain barrier (BBB) permeability, the flavones of *S. baicalensis*, baicalein and wogonin, are reported to cross the BBB after administration, which implicates their high potency for use as promising neuroprotective agents.^16^, ^17^ However, it is poorly understood whether *S. baicalensis* can attenuate reactive astrogliosis and its associated neuronal dysfunction through MAO‐B‐mediated aberrant GABA. Here, our study aims to investigate the potential anti‐astrogliosis role of *S. baicalensis* via MAO‐B inhibition using in vitro reactive astrocytes and an in vivo mouse model of neuroinflammation induced by lipopolysaccharide (LPS), a bacterial endotoxin found in gram‐negative bacteria.

## 2 MATERIALS AND METHODS

### 2.1 Preparation of *S. baicalensis* extract

We obtained the dried roots of *S. baicalensis* cultivated in Hebei Province of China. The taxonomic identification and authentication of the S. *baicalensis* sample was conducted by Prof. Hocheol Kim. The plant sample was extracted twice at 70°C for 3 h with 10 volumes of 30% (v/v) ethanol in distilled water. The liquid extract was cooled at room temperature, filtered, and concentrated under reduced pressure using a rotary evaporator, and freeze‐dried to yield a powder. The quantity of dried roots of *S. baicalensis* was 18.2 g and we obtained 8.5 g of freeze‐dried extract (extraction yield 46.70%). Voucher specimens of plant raw materials were deposited in the Herbarium of the College of Korean Medicine, Kyung Hee University (no. 15071405).

### 2.2 Experimental animals

Twenty‐seven C57BL/6J mice (male, 11–12 weeks old) were purchased from RaonBio Co. (Gyeonggi‐do, Korea). C57BL/6J species was chosen because it is widely used for modeling neuroinflammation by LPS injection.^18^ All animal experiments were carried out according to the directives of the Animal Care and Use Committee of the Korea Institute of Science and Technology (Seoul, Korea). All mice were housed in a room with automatically controlled temperature and humidity under the normal light–dark (12–12 h) cycles. For immunohistochemistry, 4 mice were used for each group (control, LPS + veh, and LPS + Extract). For slice electrophysiology, 3 mice were used for each group (control, LPS + veh, LPS + Baicalin, LPS + Baicalein, and LPS + Extract).

### 2.3 In vivo drug administration

LPS (500 μg/kg/day) and natural compounds were intraperitoneally (i.p.) injected into the mice every day for seven consecutive days. LPS (500 μg/kg/day) was reported to induce neuroinflammation and cognitive impairment in mice.^18^ Baicalin (Sigma, 572,667; 95% purity; 50 mg/kg/day), baicalein (Sigma, 465,119; 98% purity; 5 mg/kg/day), and extract (300 mg/kg/day) were intraperitoneally administered 1 h prior to the LPS injection. The dose of 300 mg/kg/day for the extract was determined based on the previous studies.^19^, ^20^ The dose of 50 mg/kg/day for baicalin was also determined based on the previous studies.^21^, ^22^ The dose of baicalein was determined based on the approximate composition ratio (baicalin:baicalein, 10:1) in the extract of *S. baicalensis*. Saline (0.9% NaCl) and 0.5% DMSO were used as controls for LPS and natural compounds, respectively.

### 2.4 High‐performance liquid chromatography (HPLC) analysis

The main components responsible for the pharmacological actions of *S. baicalensis* are flavonoids, including baicalin, baicalein, and wogonin. The contents of baicalin, baicalein, and wogonin in *S. baicalensis* extract were quantified using a Waters e2695 separations module (MA, USA) equipped with a Waters 2707 autosampler, a Waters 1525 pump, and a Waters 2998 photodiode array detector. The separation was achieved using a SunFire™ C18 column (5 μm particle size, 250 × 4.6 mm i.d.; Waters) maintained at 30°C with a flow rate of 1 mL/min. The mobile phase consisted of 1% (v/v) phosphoric acid in distilled water (A) and acetonitrile (B). The gradient elution was set as follows: 5%–50% B at 0–60 min, 50%–70% B at 60–61 min, 70%–5% B at 61–63 min, and 5%–5% B at 63–68 min. The injection volume was 10 μL. The detector wavelength for quantification was set at 254 nm, and a 3D chromatogram of UV absorption was acquired.

### 2.5 In vitro baicalin and baicalein treatment

Mouse cortical primary astrocytes derived from C57BL/6J mice P1 pups were cultured in Poly‐D‐Lysin (PDL) coated 35 mm dish in the growth media containing 10% fetal bovine serum (Gibco, 10082‐147), 10% horse serum (Gibco, 26050‐088), and 1% penicillin/streptomycin (Gibco, 15140‐122) in DMEM (Corning, 10‐013‐CV). Baicalin and baicalein were dissolved in DMSO and diluted (1:1000) in the growth media to adjust their concentration at 10, 50, and 100 μM. Primary cortical astrocytes between DIV 6 to 8 were pretreated with baicalin (10, 50, and 100 μM) or baicalein (10, 50, and 100 μM) for 60 min, followed by co‐treatment with LPS (50 ng/mL) and IFNg (10 ng/mL) for 24 h to stimulate their reactivity. The control group underwent pretreatment with DMSO only, at a final concentration of 0.1% in the growth media. To test the effect of baicalin and baicalein on normal astrocytes, primary cortical astrocytes were treated with baicalin (50 μM) or baicalein (50 μM) without the addition of LPS and IFNg. To examine the possible regional diversity in alterations of reactive astrocyte‐associated genes upon LPS and IFNg treatment, we prepared both hippocampal and cortical primary astrocytes and treated LPS (50 ng/mL) and IFNg (10 ng/mL) for 24 h to stimulate their reactivity.

### 2.6 RNA extraction and quantitative real‐time PCR (qRT‐PCR)

Total RNA was extracted from primary astrocytes by RNA‐spin™ Total RNA Extraction Kit (Intronbio, 17221) according to the manufacturer's protocol. A total of 250 ng of total RNA was synthesized into complementary DNA by SuperScript™ III First‐Strand Synthesis System (Invitrogen, 18080‐051). qRT‐PCR was conducted by QuantStudio real‐time PCR system using SYBR Green (Applied Biosystems, 4367659) with the following primer pairs: *Gapdh*_F: ACC CAG AAG ACT GTG GAT GG; *Gapdh*_R: ACA CAT TGG GGG TAG GAA CA; *Maob*_F: ACT GGT ACG TCT CAC CAA AGA A; *Maob_*R: GGC TGA CGT AGA ACC CTT CC; *Serpina3n*_F: ATC TCC ACC GAC TAC AGC CT; *Serpina3n*_R: TGT GGA CCA CCT GAG AGA CT; *C3*_F: AAG CAT CAA CAC ACC CAA CA; *C3*_R: CTT GAG CTC CAT TCG TGA CA; *Lcn2*_F: ATG TCA CCT CCA TCC TGG TC; *Lcn2*_R: AAA ATA CCA TGG CGA ACT GG; *Odc1*_F: CGT CAC TCC CTT TTA CGC AG; *Odc1*_R: AGA TAA CCC TCT CTG CAG GC; Each sample was analyzed by the 2^−∆∆Ct^ method. In detail, the difference in Ct values (ΔCt) between the gene of interest (GOI) and the internal control gene (Gapdh) was calculated using the formula ΔCt = Ct_GOI_−Ct_Gapdh_. Subsequently, the difference in ΔCt values (ΔΔCt) between the treatment group and the control group was determined through the calculation ΔΔCt = ΔCt_Treatment group_−ΔCt_Control group_. The qPCR was performed with three or four biologically independent replicates. *Gapdh* expression was used for normalization.

### 2.7 Immunofluorescence (IF)

After 7‐day co‐treatment of LPS and natural compounds, the mice were perfused with saline and 4% paraformaldehyde for IF. A total of 30 μm‐thick coronal brain slices (AP −2.0 and −2.5 mm from bregma) were obtained using cryomicrotome (Thermo Fisher). IF was conducted as previously reported,^6^ with the following primary antibodies: anti‐GFAP (chicken polyclonal, 1:1000; MilliporeSigma, AB5541), anti‐GABA (guinea pig polyclonal, 1:500; MilliporeSigma, AB175) and anti‐MAO‐B (rabbit polyclonal, 1:1000; Abcam, AB137778). Confocal images (60x) were obtained with a multiphoton laser scanning microscope (Nikon A1R, Tokyo, Japan) and analyzed by Imaris 9.3 software.

### 2.8 Astrocyte morphology analysis

Astrocyte morphology was analyzed by Sholl analysis using Neuroanatomy plugins in Fiji 2.9 software.^23^ The number of cells analyzed was 74, 105, and 59 from four mice of CTRL, LPS + Veh., and LPS + Extract groups, respectively (Table 1). The starting radius and radius step size were set by 2 μm and primary branches were inferred from the starting radius.

**TABLE 1:** Number of cells analyzed per mice for Sholl analysis.

Number of Cells (Sholl analysis)	Mouse 1	Mouse 2	Mouse 3	Mouse 4	Total
CTRL	12	30	15	17	*N* = 74
LPS + Veh	38	29	17	21	*N* = 105
LPS + Extract	10	16	10	23	*N* = 59
Number of cells analyzed per mouse	60	75	42	61	

### 2.9 MAO‐B assay

The 50% inhibitory concentration of each compound for MAO‐B enzyme activity was examined in vitro. The compounds baicalin, baicalein, and wogonin stock solutions were prepared in DMSO. MAO‐B assay was performed as previously reported with slight modification.^5^ In sample wells, 0.5 μg of Human recombinant MAO‐B (Sigma, M7441) was diluted in 100 μL of 50 mM sodium phosphate buffer (pH 7.4) and incubated with various concentrations of baicalin, baicalein, and wogonin (final concentrations ranging from 0.1 nM to 100 μM) at 37°C for 1 h. Selegiline (MAO‐B inhibitor) was added in the control wells. Due to the poor solubility of baicalin and baicalein at 100 μM concentration in the reaction buffer, we could observe the precipitates and could not obtain the correct optical absorbance at this concentration. Therefore, we constrained the bottom value as a shared value for all data sets to calculate IC_50_. Although there is a potential concern regarding the accuracy of the calculated IC_50_ value for baicalein, it is noteworthy that our calculated value (21.01 μM) closely aligns with a previously reported value of 22.1 μM.^24^

### 2.10 Slice electrophysiology for tonic GABA current measurement

Tonic GABA measurement with acute brain slices was conducted as previously reported.^3^ In detail, the mice were decapitated under the anesthesia with 2.5% isoflurane. The brains were removed from the skull and placed in an ice‐cold oxygenated (95% O_2_ and 5% CO_2_) artificial cerebrospinal fluid (ACSF; 130 NaCl, 24 NaHCO_3_, 3.5 KCl, 1.25 NaH_2_PO_4_, 1 CaCl_2_, 3 MgCl_2_ and 10 glucose (in mM), pH 7.4). Transverse hippocampal slices of 350–400 mm thickness were cut with a vibrating microtome (Neo Linear Slicer MT, Dosaka). The slices were stored in a submerged chamber with extracellular ACSF solution [126 mM NaCl, 24 mM NaHCO_3_, 1 mM NaH_2_PO_4_, 2.5 mM KCl, 2.5 mM CaCl_2_, 2 mM MgCl_2_, and 10 mM d‐(+)‐glucose (pH 7.4)] at room temperature for at least 1 h before recording.

Visually guided whole‐cell patch recordings were obtained from CA1 pyramidal neurons in the voltage clamp configuration using a MultiClamp 700B amplifier (Molecular Devices) and a borosilicate patch pipette of 5–8 MΩ resistance which was filled with internal solution [135 CsCl, 4 NaCl, 0.5 CaCl_2_, 10 HEPES, 5 EGTA, 2 Mg‐ATP, 0.5 Na2‐GTP, 10 QX‐314, pH adjusted to 7.2 with CsOH (278–285 mOsmol)]. All neurons included in this study had a resting membrane potential below −55 mV, had an access resistance in the range of 20–60 MΩ, and showed only minimal variation in these parameters during the recording period. Electrical signals were digitized and sampled at 50‐ms intervals with Axon Digidata 1550B (Molecular Devices; USA) using pCLAMP 10.2 software. Data were filtered at 2 kHz.

Before measuring the tonic current, the baseline current was stabilized with D‐AP5 (50 μM) and CNQX (20 μM). When the current was stabilized, 100 μM bicuculine was administered for tonic GABA measurement. The amplitude of tonic GABA was calculated by the baseline shift after bicuculine treatment using Clampfit 11 software. The frequency and amplitude of spontaneous inhibitory post‐synaptic currents (sIPSCs) before bicuculline administration were also detected and measured by Clampfit 11 software.

### 2.11 Statistical analysis

Statistical analyses were performed using Prism 9 (GraphPad Software, Inc.). For two group comparisons, a two‐tailed Student's *t*‐test was used. For multiple comparison tests, one‐way ANOVA or two‐way ANOVA with Tukey's or Dunnett's multiple comparisons test was used. When the data is not normally distributed, as determined by the Kolmogorov‐Sminorv normality test or the Shapiro‐Wilk normality test (when *n* of each group is lower than 6), we opted for a non‐parametric equivalent statistical test. All data are presented as mean ± standard error of the mean (SEM). *p* < 0.05 was considered to indicate statistical significance throughout the study. The significance level is represented as asterisks (*p < 0.05, **p < 0.01, ***p < 0.001; ns, non‐significant).

## 3 RESULTS

### 3.1 *S. baicalensis* extract reduces reactive astrogliosis with high GABA contents in a neuroinflammation mouse model

**FIGURE 1:**
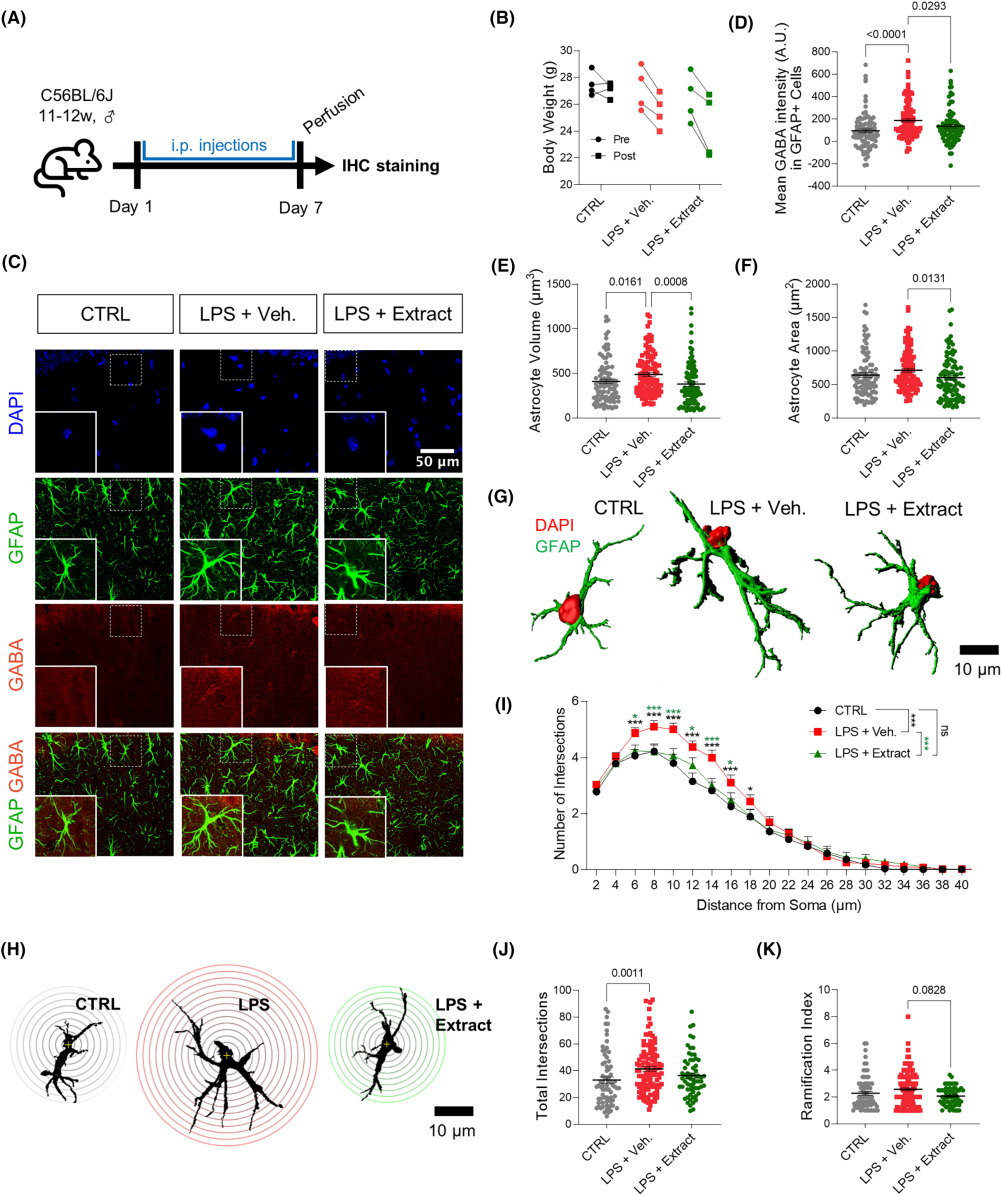
LPS‐induced reactive astrogliosis with augmented astrocytic GABA in the CA1 hippocampus is reduced by *Scutellaria baicalensis* extract treatment. (A) Schematic diagram of in vivo experiment. After pretreatment with S. *baicalensis* extract (300 mg/kg/day), male C57BL/6J mice were given daily injections of LPS (500 μg/kg/day) for 7 days. (B) Body weight changes after treatment. (C) Representative confocal images of GFAP and GABA in the CA1 hippocampus (*N* = 4 mice, and 2 slices from each mouse, for CTRL, LPS + Veh., and LPS + Extract group). Quantitative graphs for (D) mean GABA intensity in GFAP+ cells, (E) astrocyte volume, and (F) astrocyte surface area (for C–F, *n* = 96, 109, and 94 for CTRL, LPS + Veh., and LPS + Extract group). (G) Representative three‐dimensional (3D)‐rendered astrocytes. (H) Representative Sholl analysis images for GFAP+ cells. Starting radius; 2 μm, Interval of each concentric circle; 2 μm. (I) The number of intersections between concentric circles and GFAP+ cells and (J) the total number of intersections (*n* = 74, 105, and 59 for CTRL, LPS + Veh., and LPS + Extract group). (K) Ramification index of GFAP+ cells. Error bars represent means ± SEM. *p < 0.05, **p < 0.01, ***p < 0.001, ****p < 0.0001, ns, non‐significant. Statistical significance was assessed by Kruskal‐Wallis test with Duun's multiple comparisons test (D–F, J, K), or two‐way ANOVA with Tukey's multiple comparison test.

To test if LPS, a widely utilized chemical to induce extensive neuroinflammation, causes excessive astrocytic GABA synthesis, we intraperitoneally injected LPS (500 μg/kg/day) for 7 days (Figure 1A). LPS‐treated mice showed a slight reduction in body weight compared to the control group (Figure 1B). On the other hand, the ethanolic extract of *S. baicalensis* did not affect the body weight (Figure 1B). To examine the expression levels of GABA in the hippocampal astrocytes, we performed immunohistochemistry with antibodies against glial fibrillary acidic protein (GFAP) and GABA. We found that astrocytes showed significantly increased GABA contents (Figure 1C,D; *p* < 0.0001) and hypertrophy with increased cellular volume and surface area in the CA1 stratum radiatum of the hippocampus (Figure 1E–G). Additionally, Sholl analysis showed that LPS treatment significantly increased the ramification, which was evidenced by an increase in the number of intersections per unit length from the soma (*p* < 0.001) and total intersections (*p* = 0.0011) (Figure 1H–K).

Next, we tested whether *S. baicalensis* can attenuate the morphological changes of astrocytes and the excessive GABA contents in the hippocampal CA1 stratum radiatum layer of the LPS model. *S. baicalensis* extract (300 mg/kg/day, i.p.) was treated every day an hour prior to LPS administration. We found that *S. baicalensis* extract treatment significantly reduced the GABA intensity in GFAP‐positive astrocytes (Figure 1C,D; *p* = 0.0293). The extract also significantly attenuated astrocyte hypertrophy (Figure 1E–G; volume, *p* = 0.0008; area, *p* = 0.0131). Additionally, it also significantly reduced the number of intersections per unit length from soma (*p* < 0.001) and ramification index (*p* = 0.0828) (Figure 1H–K). These results indicate that *S. baicalensis* treatment is effective for reducing LPS‐induced reactive astrogliosis with high GABA contents.

### 3.2 Baicalin, baicalein, and wogonin are the major compounds of *S. baicalensis* extract

**FIGURE 2:**
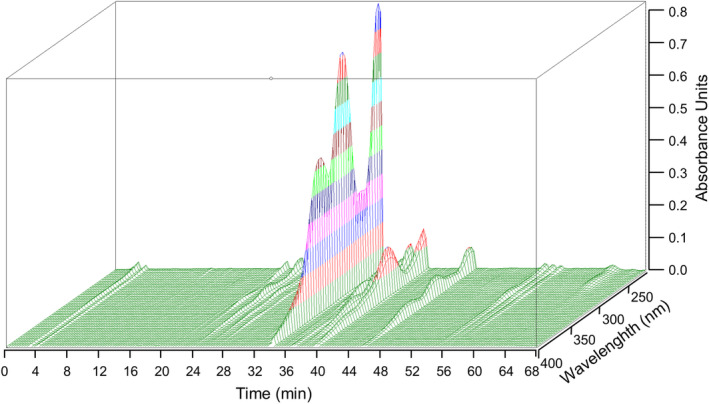
3D‐HPLC chromatogram of *Scutellaria baicalensis* extract. The three axes represent time, wavelength, and absorbance units, respectively. The values on the axis of the absorbance unit are differentiated by colors at intervals of 0.1 absorbance unit. Baicalin, baicalein, and wogonin were the major components in the extract.

We conducted HPLC analysis with ethanolic extract of *S. baicalensis*. Our three‐dimensional HPLC chromatogram analysis showed the distinct peaks of three marker compounds, baicalin, baicalein, and wogonin, in the extract (Figure 2). The retention times of baicalin, baicalein, and wogonin detected at 254 nm were 33.89, 45.71, and 55.09 min, respectively. The contents of baicalin, baicalein, and wogonin were 184.52, 12.22, and 3.13 mg/g, respectively. Baicalin, which is most abundant in *S. baicalensis* extract, showed high UV absorption at wavelengths of 214.6, 277.3, and 316.6 nm.

### 3.3 Baicalin and baicalein inhibit MAO‐B enzyme activity

**FIGURE 3:**
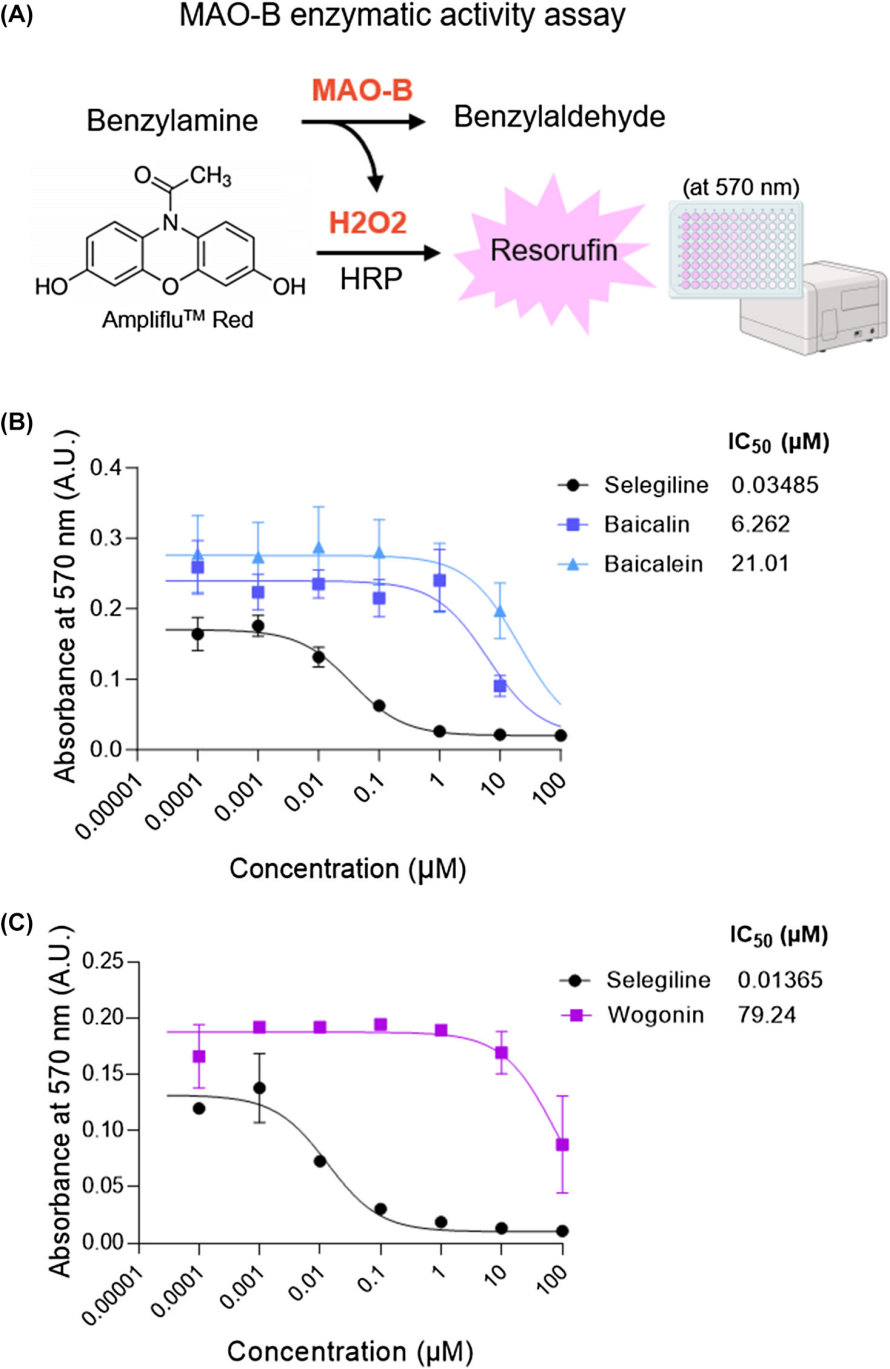
Baicalin and baicalein, the two major compounds of *Scutellaria baicalensis*, have the potential for MAO‐B inhibition. (A) Schematic diagram of MAO‐B enzyme activity assay. Created with Biorender.com. (B, C) Dose‐dependent effect of MAO‐B inhibition effect and the IC_50_ values of baicalin, baicalein, and wogonin.

To examine the MAO‐B inhibition activity of each compound, we performed an in vitro MAO‐B enzyme activity assay with purified human MAO‐B enzyme and the commercial compounds whose purities were confirmed by HPLC (Figure 3A). Selegiline, a selective irreversible inhibitor of MAO‐B, was used as a positive control. We observed that baicalin and baicalein showed potential MAO‐B inhibition effects with IC_50_ values of 6.262 and 21.01 μM, respectively (Figure 3B). On the other hand, wogonin showed relatively high IC_50_ (79.24 μM) (Figure 3C), indicating a less potent MAO‐B inhibition effect. Our findings suggest that baicalin and baicalein are the key compounds for attenuating reactive astrogliosis and the associated astrocytic GABA synthesis through MAO‐B inhibition.

### 3.4 Baicalin and baicalein down‐regulate astrogliosis‐related genes

**FIGURE 4:**
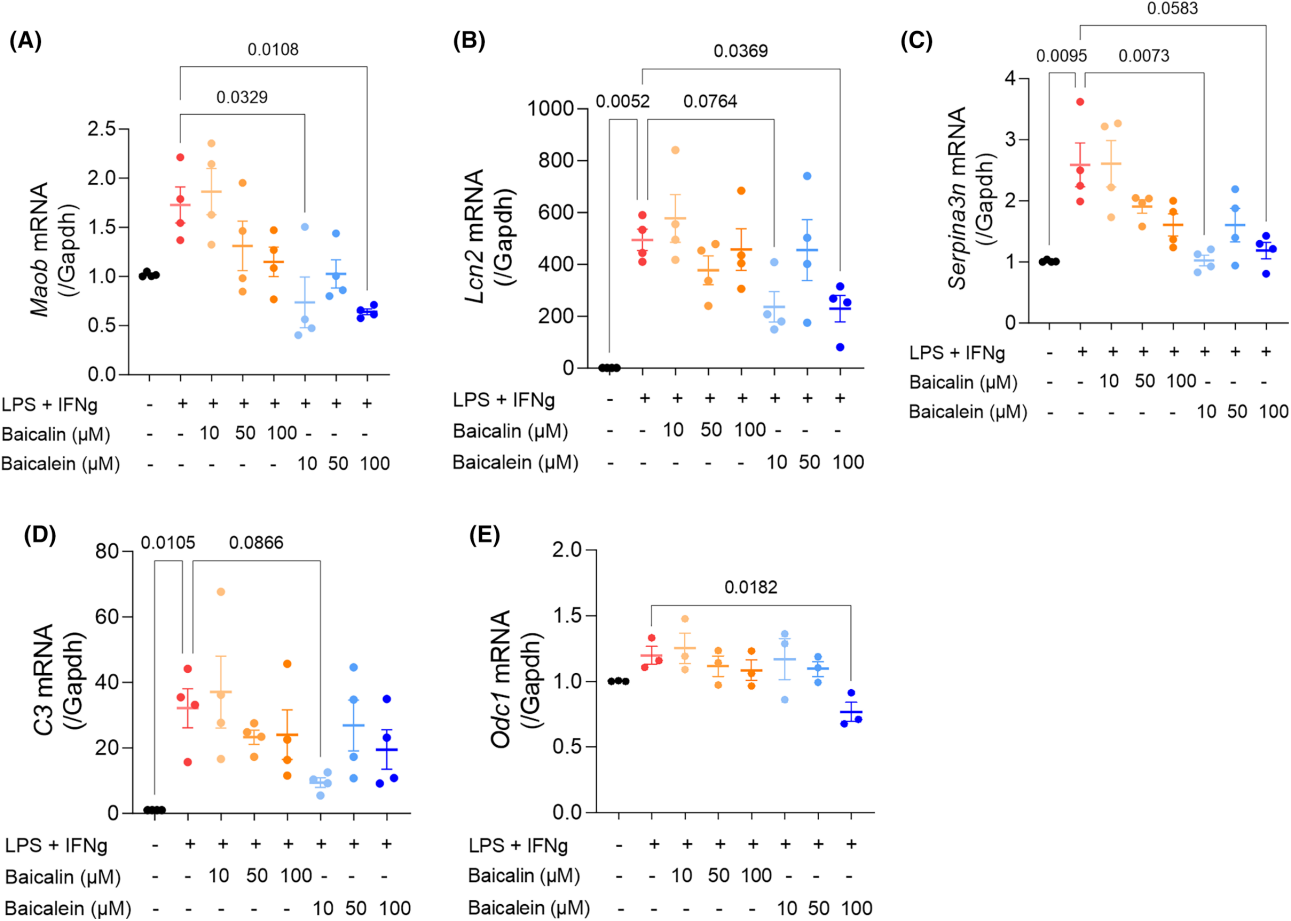
A total of 24 h in vitro treatment of baicalin and baicalein in IFNg/LPS‐induced reactive astrocytes showed downregulation of reactive astrogliosis‐related genes in a dose‐dependent manner. (A) Relative mRNA expression level of *Maob* from primary mouse cortical astrocytes incubated with baicalin (10, 50, and 100 μM) or baicalein (10, 50, and 100 μM) for 60 min, prior to IFNg (10 ng/mL) and LPS (50 ng/mL) treatment for 24 h. (B) Relative mRNA expression level of *Lcn2*. (C) Relative mRNA expression level of *Serpina3n*. (D) Relative mRNA expression level of *C3*. (E) Relative mRNA expression level of *Odc1*. Error bars represent means ± SEM. *p < 0.05, **p < 0.01, ***p < 0.001. Statistical significance was assessed by Kruskal‐Wallis test with Duun's multiple comparisons test (A, B) or One‐way ANOVA with Dunnett's multiple comparisons test (C, D, E). Four biologically independent replicates were tested.

Then, we performed a 24 h in vitro treatment of baicalin (10, 50, and 100 μM) or baicalein (10, 50, and 100 μM), the two major MAO‐B‐inhibiting compounds, into reactive astrocytes which were induced by LPS (50 ng/mL) and IFNg (10 ng/mL), based on previous reports.^25^, ^26^ Since primary cultured astrocytes do not naturally secrete IFNg, co‐incubation with both LPS and IFN‐γ may more efficiently simulate an inflammatory response compared to using LPS alone. We found that primary cortical astrocytes treated with LPS/IFNg showed a significant increase in the mRNA expression of *Maob* and other astrogliosis markers including lipocalin 2 (*Lcn2*) (*p* = 0.0052), serpin family A member 3 N (*Serpina3n*) (*p* = 0.0095), and complement component 3 (*C3*) (*p* = 0.0105) (Figure 4A–D). The LPS and IFNg‐induced alterations in the expression levels of *Lcn2, C3, Serpina3n*, and *Odc1* in the cortical astrocytes were comparable with those in the hippocampal astrocytes, while these reactive astrocyte‐related genes exhibited notably higher increases in the cortical astrocytes compared to the hippocampal astrocytes (Figure S1). We further observed that baicalein significantly and dose‐dependently reduced the elevated expression of *Maob* (Figure 4A; *p* = 0.0329 and 0.0108 for 10 and 100 μM of baicalein, respectively) and *Serpina3n* (Figure 4C; *p* = 0.0073 and 0.0583 for 10 and 100 μM of baicalein, respectively), while *Lcn2* and *C3* also showed a decreasing trend (Figure 4B,D). Baicalin also showed a dose‐dependent decrease in the mRNA expression level of all observed genes, although it was not statistically significant. Additionally, we examined the mRNA expression of ornithine decarboxylase‐1 (ODC1), a recently identified critical enzyme for astrocytic GABA synthesis. We observed the trend of elevated *Odc1* by IFNg/LPS is significantly decreased following 100 μM baicalein treatment (Figure 4E). On the other hand, baicalin and baicalein did not significantly alter the expression levels of astrogliosis‐related genes, such as *Lcn2, C3, Serpina3n*, and *Odc1* (Figure S2). Collectively, these findings indicate that both baicalin and baicalein have an anti‐astrogliosis effect, with baicalein possibly demonstrating a more advantageous anti‐astrogliosis effect than baicalin.

### 3.5 *S. baicalensis* relieves the excessive tonic inhibition of hippocampal neurons in a neuroinflammation mouse model

**FIGURE 5:**
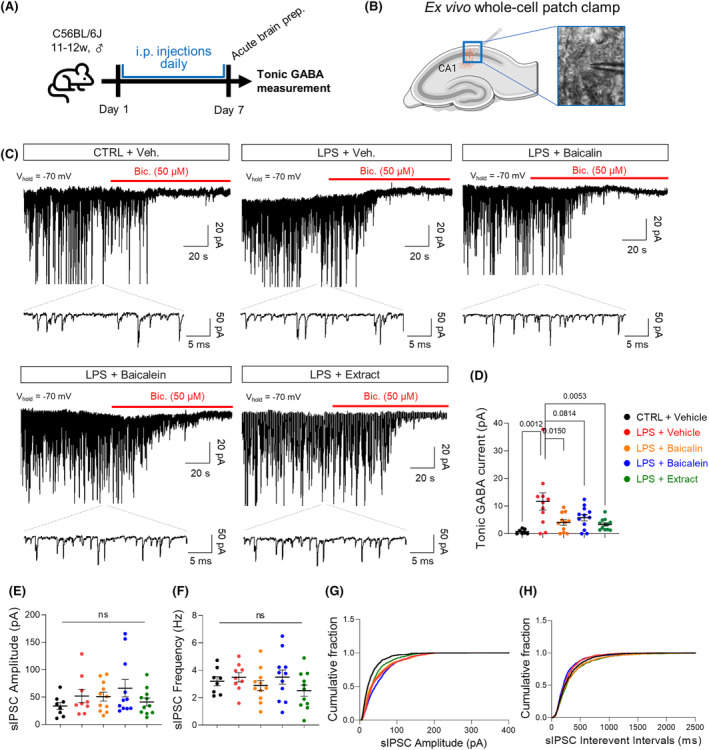
Systemic administration of *Scutellaria baicalensis* extract and its two major compounds decreases the excessive GABA‐mediated tonic inhibition of CA1 pyramidal neurons. (A) Schematic diagram of in vivo experiment. After pretreatment with *S. baicalensis* extract (300 mg/kg/day), baicalin (50 mg/kg/day), or baicalein (5 mg/kg/day), male C57BL/6J mice were given daily injections of LPS (500 μg/kg/day) for 7 days. (B) Schematic diagram of slice patch clamp. Created with Biorender.com. (C) Representative trace of GABA_A_R‐mediated current (*n* = 8, 11, 11, 12, and 12 from 3 mice for each group). (D) Tonic GABA current. (E) Average amplitude and (F) average frequency of sIPSC. Cumulative probability distributions of (G) interevent interval values and (H) amplitude of sIPSC. Error bars represent means ± SEM. *p < 0.05, **p < 0.01, ***p < 0.001, ns, non‐significant. Statistical significance was assessed by One‐way ANOVA with Tukey's multiple comparisons test (D, F) or Kruskal‐Wallis test with Duun's multiple comparisons test (E).

Next, we investigated whether *S. baicalensis* can reverse the aberrantly increased tonic inhibition of hippocampal neurons in an LPS‐induced neuroinflammation mouse model (Figure 5A). To test this, we performed an ex vivo whole‐cell patch‐clamp experiment on CA1 hippocampal pyramidal neurons and recorded the tonic GABA currents (Figure 5B). Tonic GABA current was estimated by the baseline shift caused by bicuculline, a specific blocker against the GABA_A_ receptor. We found that the LPS group showed significantly higher tonic GABA current compared to the control group (11.67 ± 3.12 pA vs. 2.09 ± 1.38 pA; *p* = 0.0012) (Figure 5C,D). However, treatment with baicalin, baicalein, and the ethanolic extract of *S. baicalensis* markedly decreased tonic GABA current (baicalin, 4.08 ± 1.01 pA, *p* = 0.0150; baicalein, 5.77 ± 1.15 pA, *p* = 0.0814; extract, 3.35 ± 0.58 pA, *p* = 0.0053) in the hippocampus of LPS‐induced neuroinflammation mouse model (Figure 5C,D). Meanwhile, spontaneous inhibitory postsynaptic currents (sIPSC) frequency and amplitude were not significantly different across the groups (Figure 5E–H). Therefore, our findings indicate that the ethanolic extract of *S. baicalensis* and its two major compounds are effective in lowering reactive astrocyte‐mediated aberrant tonic inhibition of hippocampal neuronal activity in the LPS‐induced neuroinflammation model.

## 4 DISCUSSION

Neuroinflammation is a self‐defense mechanism in the central nervous system triggered by injury, viral infection, exposure to toxins, neurodegenerative diseases, or aging. However, the prolonged neuroinflammatory responses, including reactive astrogliosis, paradoxically lead to neurodegenerative diseases such as Parkinson's disease (PD) and Alzheimer's disease (AD).^27^ Therefore, targeting neuroinflammation is crucial for treating a wide range of brain disorders associated with it.

Reactive astrocytes are known as a critical mediator of neuroinflammation‐induced neuronal dysfunction and damage.^28^ Prolonged neuroinflammation can trigger reactive astrogliosis, which has been reported as an etiology for the development of neurodegenerative diseases. Reactive astrocytes are characterized by significant morphological changes, particularly hypertrophy, and proliferation.^1^ In addition to the morphology, reactive astrocytes have also characteristic transcriptional and functional properties. Particularly, accumulating lines of evidence have shown that reactive astrocytes synthesize an excessive amount of GABA through the enzymatic action of a mitochondrial enzyme, MAO‐B.^3^, ^5^, ^6^, ^7^ Excessive GABA content has been observed in multiple brain regions of various pathological or neuroinflammatory conditions, including hippocampus of AD model,^5^, ^29^, ^30^ substantia nigra of PD model,^8^ and cerebral cortex of white matter stroke model.^7^ Moreover, the excessive astrocytic GABA‐mediated tonic inhibition of neighboring neurons has been attributed as a critical pathological factor of these disorders.^3^, ^4^, ^5^, ^6^, ^7^ Therefore, MAO‐B‐mediated excessive GABA synthesis could be considered a core characteristic of reactive astrocytes as well as a key therapeutic target throughout a variety of neuroinflammatory conditions. In this regard, there have been accumulating attempts to discover natural compounds that block MAO‐B and several natural compounds have been indeed reported as potential natural MAO‐B inhibitors.^24^

Among a plethora of medicinal herbs that are used in traditional Asian medicines, *S. baicalensis* has been intensively studied for the past decades in various brain disorders, including stroke, AD, and PD, all of which are related to reactive astrocyte.^3^, ^4^, ^5^, ^7^ In detail, *S. baicalensis* and its major flavonoids, including baicalin, baicalein, and wogonin, alleviate cerebral ischemia/reperfusion injury by reducing oxidative and inflammatory responses and inhibiting neuronal apoptosis.^31^ Baicalin and its aglycon, baicalein, attenuate brain edema and blood–brain barrier disruption in animal models of intracerebral and subarachnoid hemorrhage.^32^, ^33^ Baicalin and baicalein also showed the neuroprotective effects in MPTP and 6‐hydroxydopamine‐induced mouse models of PD by inhibiting reactive astrogliosis, oxidative stress, and inflammatory response and by increasing striatal catecholamines.^34^, ^35^ Regarding AD, baicalein prevented memory deficits in the APP/PS1 AD mouse model through decreased Aβ production and inhibited tau phosphorylation.^36^ In addition, *S. baicalensis* and its flavonoids have been reported to exert protective effects in rodent models of spinal cord injury, depression, and traumatic brain injury.^13^, ^14^ Moreover, at least some of these potential therapeutic effects of *S. baicalensis* and its major compounds have been suggested to be associated with regulation of reactive astrocytes.^34^, ^35^ However, the molecular mechanism underlying how these compounds attenuate reactive astrogliosis has less been explored.

**FIGURE 6:**
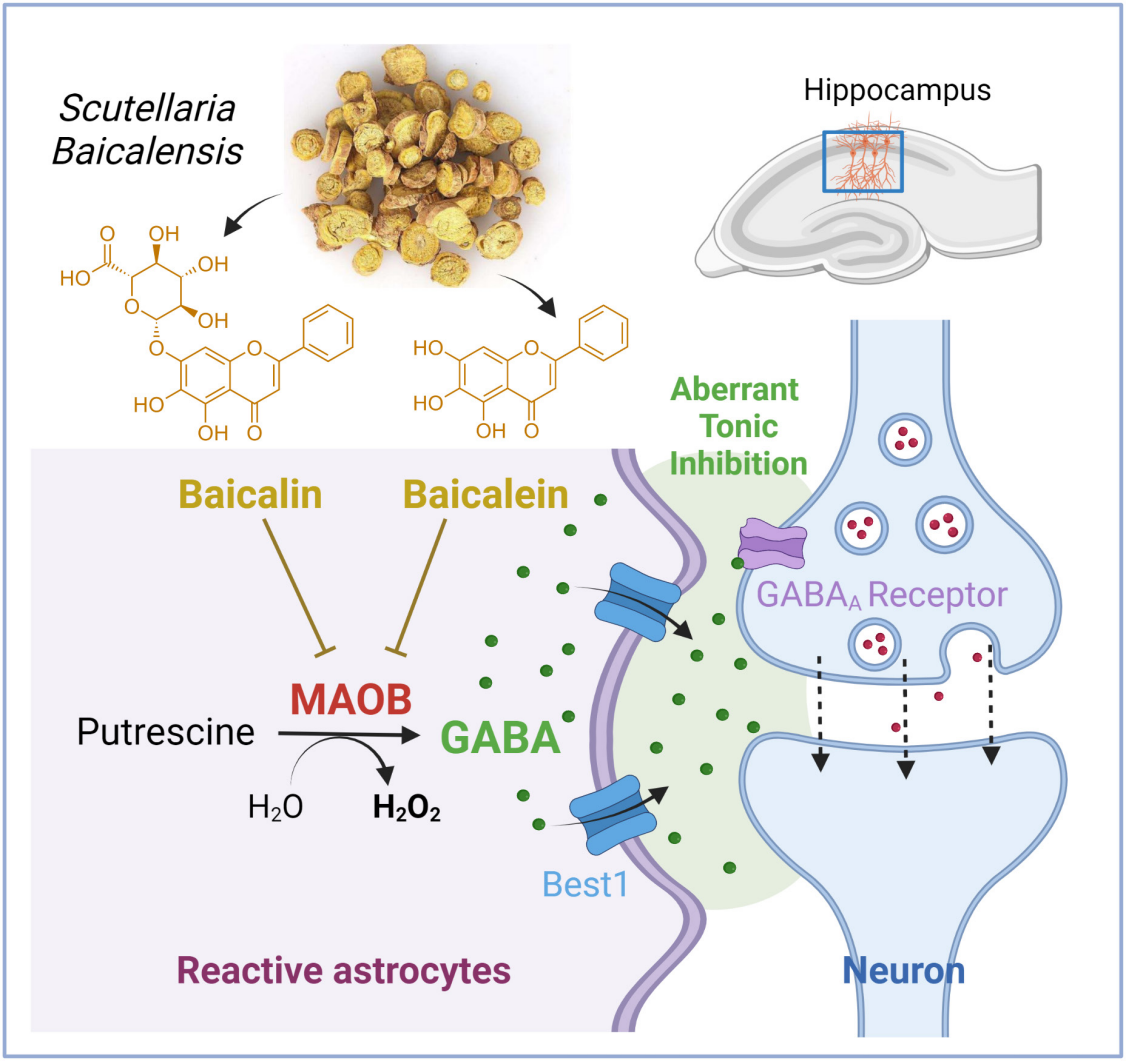
The proposed mechanism of *Scutellaria baicalensis* for astrocytic GABA‐mediated neuronal dysfunction in neuroinflammation. Baicalin and baicalein, the major components of *S. baicalensis*, inhibit the enzymatic activity of monoamine oxidase B (MAO‐B), thereby diminishing GABA synthesis in reactive astrocytes. This reduction in astrocytic GABA production leads to the alleviation of GABA_A_ receptor‐mediated tonic inhibition in hippocampal neurons.

In the current work, we clearly demonstrated that baicalin and baicalein, compounds extracted from *S. baicalensis*, can inhibit the enzymatic action of MAO‐B, leading to the significant restoration of excessive tonic inhibition of hippocampal neurons mediated by astrocytic GABA (Figure 6). We also observed a general decreasing trend in the expression levels of key genes associated with reactive astrogliosis, including *Maob, Lcn2, Serpina3n, C3*, and *Odc1*, upon treatment with baicalin and baicalein (Figure 4). To note, the efficacy of the *S. baicalensis* extract was not assessed in the study. While some genes displayed a significant reduction, others exhibited a consistent tendency towards downregulation, collectively suggesting a propensity for a general decrease in gene expression associated with reactive astrogliosis. *Lcn2* is well‐documented as a critical mediator of neuronal death induced by reactive astrocytes.^37^ *Serpina3n* and *C3*, together with *Lcn2*, are highlighted as the key genes of A1‐like neurotoxic reactive astrocytes.^38^ ODC1 is a critical enzyme for neurodegenerative disease through converting ornithine into putrescine, which is the substrate of MAO‐B for astrocytic GABA production.^4^ Meanwhile, previous studies have shown MAO‐B is a key regulator of reactive astrocyte alterations.^2^, ^5^ Therefore, our findings suggest that MAOB inhibition by baicalin and baicalein could have a potent effect on alleviating reactive astrogliosis.

The effects of the major compounds of *S. baicalensis* on MAO‐B inhibition have been investigated by a few previous studies, but the results were inconsistent. For example, an in vitro study showed the potential MAO‐B inhibition effects of baicalin (a theoretical inhibition constant (Ki) value of 170.64 nM) and baicalein (an IC_50_ value of 22.1 μM), which is comparable to our study (IC_50_ value of 21.01 μM for MAO‐B, Figure 3B),^24^ while another in vitro study reported that baicalin and baicalein have no effect on MAO‐B inhibition but wogonin has a potential for MAO‐B inhibition, showing the IC_50_ of 20.8 μM for MAO‐B.^39^ The discrepancies in the findings may be attributed to differences in experimental design, methods of compound extraction, purity of the substances, or other factors that require further investigation. Here, our study provides clear evidence that baicalin and baicalein have superior MAO‐B inhibition effects than wogonin (Figure 3). Furthermore, our results from ex vivo electrophysiology experiments underpinned their potent MAO‐B inhibition effects by demonstrating that treatment of baicalin and baicalein can significantly reduce the astrocytic GABA‐mediated tonic inhibition of neighboring neurons in the hippocampus.

The inhibition of MAO‐B can be a promising strategy for neurodegenerative disorders, as this enzyme is involved not only in GABA‐mediated neuronal dysfunction but also in H_2_O_2_‐mediated neuronal death (Figure 6). Specifically, during the production of GABA from putrescine, MAO‐B promotes the production of NH_2_ and H_2_O_2_ as byproducts, leading to an increase in toxic ammonia and nitrosative stress.^5^ Therefore, *S. baicalensis* and its major compounds, baicalin and baicalein, may have therapeutic potential for neuroprotection, which is an interesting possibility that should be explored in future research.

## 5 CONCLUSIONS

In conclusion, this study provides the first concrete evidence that *S. baicalensis*, along with its major flavonoids baicalin and baicalein, can effectively reduce reactive astrogliosis and the associated aberrant tonic inhibition of neurons in the brain by inhibiting MAO‐B. Given that aberrant tonic inhibition of neurons can lead to various neurological symptoms such as memory deficits and motor dysfunction, this medicinal herb and its natural compounds hold great promise as pharmacological candidates for various brain disorders that involve neuroinflammation.

## AUTHOR CONTRIBUTIONS

Conceptualization by M.‐H.N. and H.K. (Hocheol Kim); Methodology by J.S. and H.K.; Investigation and validation by J.C., Y.H.J., H.K. (Hyunjin Kim), E.‐B.H.; Resources by Y.‐S.K., M.‐H.N., and H.K.; Writing ‐ Original Draft by J.C., J.S., and M.‐H.N.; Writing ‐ Review & Editing by J.C., M.‐H.N., and H.K. (Hocheol Kim); Supervision by M.‐H.N., and H.K. (Hocheol Kim); Funding acquisition by M.‐H.N., and H.K. (Hocheol Kim).

## CONFLICT OF INTEREST STATEMENT

The authors have no conflicts of interest to declare.

## Supporting information


**Appendix S1.** Supporting information.

## Data Availability

The data that support the findings of this study are available from the corresponding author upon reasonable request.
