# Supplementary material for: Baicalin and baicalein from Scutellaria baicalensis Georgi alleviate aberrant neuronal suppression mediated by GABA from reactive astrocytes
Source: CNS Neurosci Ther. 2024 May 7;30(5):e14740. doi: 10.1111/cns.14740 (PMC11076983; doi:10.1111/cns.14740)
Supplement: Supplementary file 1 — Appendix S1. Supporting information. [file CNS-30-e14740-s001.docx]

Supplementary Material

**Baicalin and baicalein from *Scutellaria baicalensis* Georgi alleviate aberrant neuronal suppression mediated by GABA from reactive astrocytes**

Juyeong Cho^a^, Eun-Bin Hong^a^, Young-Sik Kim^b^, Jungbin Song^c^, Yeon Ha Ju^a^, Hyunjin Kim^a^, Hyowon Lee^a^, Hocheol Kim^c^,*, and Min-Ho Nam^a,d,e^*

includes Supplementary Figure 1 and 2


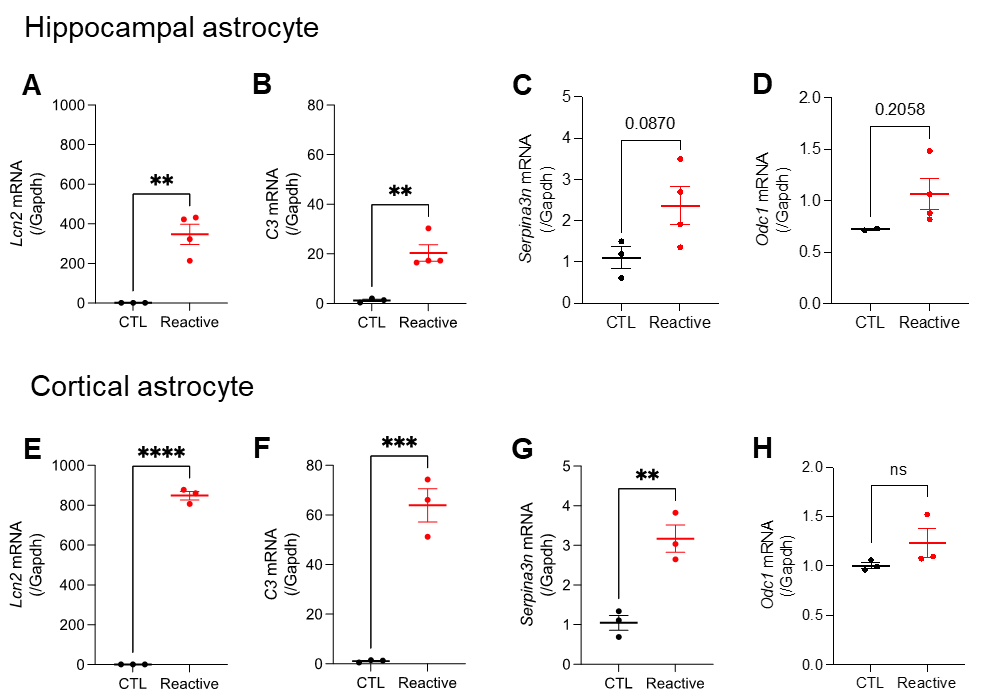


**Supplementary Figure 1. Gene alterations in hippocampal and cortical primary astrocytes following LPS/IFNg treatment**

(A-D) Relative mRNA expression level of Lcn2, C3, Serpina3n, and Odc1 from primary mouse hippocampal astrocytes incubated with IFNg (10 ng/mL) and LPS (50 ng/mL) treatment for 24 h, compared to control astrocytes. (E-H) Relative mRNA expression level of Lcn2, C3, Serpina3n, and Odc1 from primary mouse cortical astrocytes incubated with IFNg (10 ng/mL) and LPS (50 ng/mL) treatment for 24 h. Error bars represent means ± SEM. *p < 0.05, **p < 0.01, ***p < 0.001, ns, non-significant. Statistical significance was assessed by two-tailed Student’s t-test (A, C, E-H) and Mann-Whitney test (B). Three biologically independent replicates were tested except for control in (D).


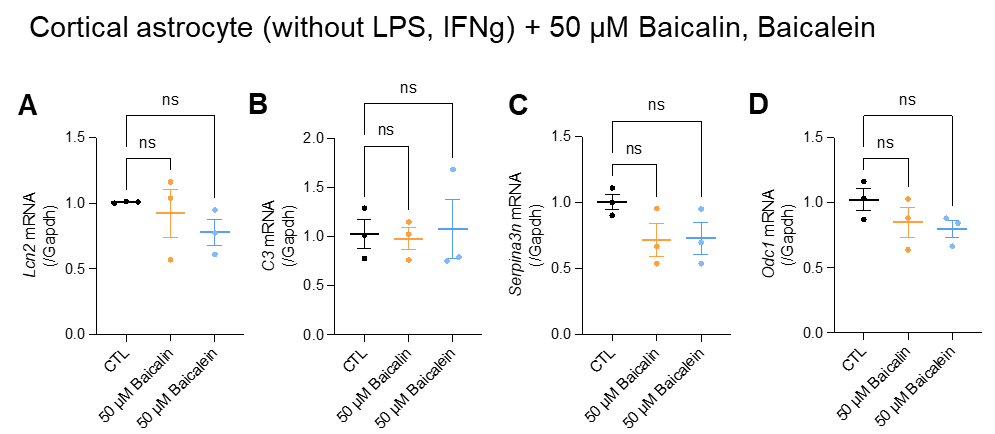


**Supplementary Figure 2. Effect of baicalin and baicalein in normal astrocytes**

(A-D) Relative mRNA expression level of Lcn2, C3, Serpina3n, and Odc1 from primary mouse cortical astrocytes incubated with baicalin (50 μM) and baicalein (50 μM) for 24 h. Error bars represent means ± SEM. ns, non-significant. Statistical significance was assessed by two-tailed Student’s t-test. Three biologically independent replicates were tested.
